# The impact of weight and race on perceptions of anorexia nervosa: a replication and extension of Varnado-Sullivan et al. (2020)

**DOI:** 10.1007/s40519-025-01748-x

**Published:** 2025-04-30

**Authors:** Nathalie Gullo, Olivia Brand, Erin Harrop, D. Catherine Walker

**Affiliations:** 1 Washington University, St. Louis, 1 Brookings Drive, St. Louis, MO 63130 USA; 2 Department of Psychology, Union College, 807 Union Street, Schenectady, NY 12308 USA; 3 Department of Social Work, University of Denver, 2148 S. High St., Denver, CO 80208-7100 USA

**Keywords:** Weight, Race, Anorexia nervosa, Atypical anorexia nervosa, Stigma, Weight stigma, Intersectionality

## Abstract

**Purpose:**

This study examined how weight and race impact mental health stigma, weight stigma, perceived need for treatment, and perceived severity of anorexia nervosa We experimentally manipulated weight and race, replicating and extending Varnado-Sullivan et al. (Eat Weight Disord 25:601–608, 2020).

**Methods:**

336 participants were recruited from Prolific. Participants self-reported pre-existing exposure to and attitudes regarding mental illness. Participants were randomly assigned to read an anorexia nervosa vignette that manipulated race (White or Black) and weight (“underweight” or “obese”). Participants self-reported attitudes about the woman in the vignette (mental health stigma), weight stigma, and perceived need for treatment and severity of the condition (mental health literacy). We hypothesized that greater mental health stigma, weight stigma, and lower mental health literacy would be present for Black and higher-weight vignettes, controlling for covariates.

**Results:**

Analyses found that only vignette weight significantly predicted mental health stigma, mental health literacy, and weight stigma; vignette race did not significantly predict mental health stigma, mental health literacy, or weight stigma. A significant Race x Weight interaction predicted weight stigma and two mental health stigma items.

**Conclusion:**

Replicating and extending Varnado-Sullivan et al. (Varnado-Sullivan et al. in Eat Weight Disord 25:601–608, 2020), we found weight-based bias for those with eating disorders, with some interactions between weight and race on weight stigma.

**Supplementary Information:**

The online version contains supplementary material available at 10.1007/s40519-025-01748-x.

## The impact of weight and race on perceptions of anorexia nervosa

Eating disorders (EDs) are severe psychological disorders with high morbidity [1] and mortality rates [1, 2]. Anorexia nervosa (AN) has the highest mortality rate of EDs, estimated at 5–7% [1]. Currently, for an individual to be diagnosed with AN in the *Diagnostic and Statistical Manual of Mental Disorders* (*5 th ed., text rev.; DSM–5-TR*) [3], an individual needs to be restricting energy intake leading to significantly low body weight. Anorexia nervosa has typically been studied in and, consequently, described as being most common among low-weight, predominantly White adolescents and young adults with higher socioeconomic status [4, 5], with relatively low population prevalence rates of just 0.6% among adults [6].

However, recent research suggests that symptoms consistent with AN are present among individuals who are not clinically “underweight” with equal or greater prevalence than AN [7–9] and among more demographically diverse groups [8, 10, 11]. Higher-weight individuals with EDs face greater stigma and often have their disordered eating and unhealthy weight-loss behaviors reinforced or deemed insignificant [12–15]. Weight bias held by the general population and medical and mental health practitioners may result in inequitable identification and treatment referrals for EDs in higher-weight individuals (e.g., [16–19]), and thus inadequate treatment.

### Atypical anorexia nervosa

Atypical AN (AAN) was first categorized as one of several syndromes diagnosed as an ‘ED not otherwise specified’ (EDNOS) in the DSM-IV and remains an ‘other specified feeding or ED’ (OSFED) in the DSM-5-TR [3]. Many individuals with AAN may not receive appropriate treatment due to lack of knowledge regarding OSFED and AAN among medical professionals and the public, and due to weight stigma [16]. Regardless of this treatment gap and stigma, the symptoms of AAN are the same as low-weight AN (e.g., energy restriction leading to significant weight loss, intense fear of weight gain, overvaluation of weight or shape), with the only difference being the requirement for those with AAN to not be at a low body weight despite significant weight loss [3]. For example, individuals who did not meet the underweight BMI criterion were not accepted to treatment programs for AN [20, 21], and many insurance companies in the U.S. still use BMI to determine coverage for individuals with EDs [22].

Atypical AN is identified less frequently and treated less urgently than AN because individuals with AAN do not have a BMI under 18.5 kg/m^2^, even though they may be significantly weight-suppressed [9, 16, 23]. Research has demonstrated evidence of weight bias even among mental health practitioners and those specializing in EDs [18, 19, 24]. For example, mental health trainees [19] and practitioners [18] were more likely to recommend fewer treatment sessions for a patient vignette described as overweight or normal weight presenting with AAN compared to the same vignette with an underweight BMI (meeting criteria for AN), even though the vignettes had identical descriptions of symptoms and pathology. Research suggests that people with AAN are presenting with the same symptoms and sometimes more severe pathology than those with AN [23, 25, 26]. Garber and colleagues [23] found that ED psychopathology was more severe in patients with AAN, potentially due to the increased weight stigma heavier people face. Furthermore, individuals with AAN often experience severe weight loss, malnutrition, and comorbid diagnoses, experiencing severe medical complications on par with that of low weight AN [16, 26, 27]. Taken together these findings indicate that AAN is an ED with high medical and psychological severity, meriting treatment, even when weight loss or malnutrition is not overtly evident from the person’s current BMI.

Higher-weight individuals with restrictive EDs are more likely to receive positive feedback and affirmations about weight loss from friends, family members, and healthcare providers [16]. This often leads the disorder to be viewed as “health promoting,” reducing treatment-seeking; the disorder also often goes unrecognized by healthcare providers even when individuals seek treatment [13, 14]. For example, using data from a large longitudinal study of children and adolescents who met criteria for an ED at any point from 1996 to 2013 (*N* = 1,237), researchers found that those with BMIs ≥ 30 kg/m^2^ were 85% less likely to receive treatment regardless of the specific ED diagnosis [8]. Taken together, evidence suggests that weight stigma plays a role in the underdiagnosis of EDs, especially in higher-weight people [8].

### Weight stigma and eating disorders

Weight stigma results in many consequences for larger-bodied people, including ostracization, employment bias, and prejudice in healthcare settings [28–30], among others. Weight stigma has also been demonstrated in mental health and ED settings. Ebneter and Latner [31] examined within-groups differences between five vignettes describing a 19-year-old woman with AN, bulimia nervosa with normal weight, binge eating disorder with obesity, no psychological diagnosis with obesity, or major depressive disorder (MDD) with normal weight^1^. Compared to MDD, women with EDs were viewed as having greater control of their disorder. Furthermore, of all the EDs, the woman with BED with obesity was rated as the most responsible for her disorder. When compared to the woman described as having obesity without an ED, the woman with BED was more stigmatized. These findings were replicated with vignettes of both women and men [14]. Thus, binge-eating and having a higher weight may be seen as an issue of self-control compared to other EDs [31]. Similar results were seen comparing six vignettes that described a woman with bulimia nervosa, manipulating her weight and symptom severity across conditions; higher weight status but not symptom severity was associated with more negative perceptions, perception of the issue as less severe, and lower perceived need for treatment [12]. Overall, findings demonstrate the impacts of weight stigma in recognizing EDs and recommending proper treatment both among laypersons (e.g., [15, 31]), and among mental health practitioners [17–19].

### Treatment disparities in people of color

Most early ED research focused on eating and body image concerns more prevalent among White women and was conducted among predominantly White female samples. This likely both reflects and reifies the stereotype that EDs primarily affect young, affluent White women, reinforcing racial bias in clinical training, research, and sampling [32]. Therefore, AN and many other EDs are underdiagnosed in racial and ethnic minority (REM^2^) groups [2, 33], with REM individuals half as likely to receive a diagnosis or treatment for their ED compared to White counterparts [2].

Early literature on EDs across different racial/ethnic groups posited that women from REM groups were less likely to develop certain EDs assuming that Black and Latinx communities in particular were less likely to internalize Eurocentric beauty standards and were more accepting of larger bodies (e.g., [5, 34]). However, the suggestion that REM women are not as vulnerable to developing EDs due to cultural differences may be misleading, contributing to further health inequities for REM women. Statistics on EDs in REM women may be underreported; some research suggests that many REM women may wait for symptoms to get worse before seeking treatment, may not have equal access to health care or treatment, may use different language to communicate symptoms, and may be faced with biases and racism in healthcare that dissuade them from seeking treatment [32, 33]. Cachelin et al. [35] found that among 61 ethnically diverse women (31% White) from an urban community who were diagnosed with an ED by the research team, although 85.2% wanted ED treatment, only 57% made treatment contact, and, of those, only 14% received ED treatment. Notably, of the treatment-seeking participants in this study, 65.7% were prescribed treatment for weight loss, and only one woman with BED (5.9%) received ED treatment [35], suggesting both weight and racial bias may explain why REM individuals are less likely to receive ED diagnoses and treatment. Racial discrimination may be a risk factor for ED symptomatology, with REM individuals endorsing higher levels of restriction symptoms in a mixed-race sample of college students [36]. Furthermore, more REM individuals have a history of AAN than AN [37]. Thus, it is likely that the intersection of racial and weight bias may lead to underdiagnosis of EDs among higher-weight REM individuals, in particular. Research needs to be cognizant of the prevalence of EDs in different racial/ethnic groups and of historic bias and underdiagnosis to ensure accurate diagnosis and treatment [38]; yet to date, minimal research has examined racial bias in identifying cases of AN.

### Current study

While previous research has examined treatment inequality across AN and AAN [9, 20, 26, 27, 39], fewer studies have examined weight stigma in higher-weight persons with AAN compared to “underweight” individuals with AN [17–19, 40]. Further, to the best of our knowledge, no research has examined the impacts of weight stigma *and* race across AN/AAN. This paper replicates and extends previous research by Varnado-Sullivan and colleagues [41], who compared mental health stigma and mental health literacy among undergraduate participants randomly assigned to read a vignette describing a woman with AN, MDD, and a control vignette describing a woman who was lonely. Varnado-Sullivan et al. [41] found significant effects of vignette condition on both mental health stigma and mental health literacy scales, with the woman with AN rated as most enthusiastic, driven, disciplined, least lazy, and least attractive, and AN was rated as more serious, distressing, most difficult to recover from, most in need of treatment, and more of a problem, but associated with the most perceived shame when seeking treatment. The current study extends this research by examining how weight stigma and race influence mental health stigma, mental health literacy (perceived severity and need for treatment), and weight stigma across different conditions of AN/AAN. Consistent with previous research stigmatizing women with ED in larger bodies the most [14, 15, 31], it was hypothesized that the women in the AAN condition described as being in the “obese” weight range would be viewed as more in control of their disorder, less in need of treatment, having a less serious condition, and associated with higher mental health stigma. Consistent with previous research [32, 42, 43], it was also hypothesized that the Black women in both the AN and AAN conditions would experience more mental health stigma than the White women in both weight conditions, but that the Black woman with AAN described as being in the “obese” weight range would experience more mental health and weight stigma than the Black “underweight” woman with AN or White woman with AAN. Lastly, we examined these questions controlling for pre-existing experiences with and attitudes towards individuals with mental illness.

## Method

### Participants

Participants were recruited from Prolific and compensated for participation. Inclusion criteria were ≥18 years old and U.S. location. Survey duration, bot check, and attention check questions alongside appropriate responses to height, weight, and open-ended questions were used to screen for invalid responses. Data were collected from 340 participants; 4 were removed due to incomplete/invalid data, with 336 participants included in the analysis, *M*(*SD*)_Age_ = 35.57 (12.93; range; 18–74) years and *M*(*SD*)_BMI_ was 27.73(8.07) kg/m^2^, range: 15.2–59.3. Participants’ demographic data, separated by condition, are presented in Table 1.

**Table 1:** Participant demographic characteristics by condition

Variable	AAN, white	AN, black	AAN, black	AN, white	MDD control	*χ*^2^	*p*
	*n* (%)	*n* (%)	*n* (%)	*n* (%)	*n* (%)		
Gender							
Cisgender woman	38 (17.1%)	44 (19.8%)	51 (23.0%)	46 (20.7%)	43 (19.4%)	22.6	0.31
Cisgender man	24 (27.9%)	16 (18.6%)	14 (16.3%)	19 (22.1%)	13 (15.1%)		
Non binary/third gender	3 (30%)	3 (30%)	2 (20%)	0 (0%)	2 (20%)		
Transgender woman	1 (33.3%)	0 (0%)	0 (0%)	0 (0%)	2 (66.7%)		
Transgender man	0 (0%)	3 (50%)	1 (16.7%)	1 (16.7%)	1 (16.7%)		
Prefer not to say	1 (11.1%)	2 (22.2%)	0 (0%)	3 (33.3%)	3 (33.3%)		
Race							
White	40 (19.5%)	42 (20.5%)	52 (25.4%)	38 (18.5%)	33 (16.1%)	31.6	0.14
Black/African American	8 (16%)	4 (8%)	6 (12%)	17 (34%)	15 (30%)		
Latin American	5 (21.7%)	5 (21.7%)	4 (17.4%)	4 (17.4%)	5 (21.7%)		
American Indian or Alaskan Native	0 (0%)	1 (50%)	0 (0%)	0 (0%)	1 (50%)		
Asian	10 (27%)	10 (27%)	4 (10.8%)	8 (21.6%)	5 (13.5%)		
Native Hawaiian or Pacific Islander	0 (0%)	1 (100%)	0 (0%)	0 (0%)	0 (0%)		
Other	4 (22.2%)	5 (27.8%)	2 (11.1%)	2 (11.1%)	5 (27.8%)		
Education							
Some high school	0 (0%)	0 (0%)	1 (33.3%)	0 (0%)	2 (66.7%)	27.9	0.11
High school	5 (10.2%)	9 (18.4%)	6 (12.2%)	14 (28.6%)	15 (30.6%)		
Some college	18 (15.3%)	27 (22.9%)	27 (22.9%)	21 (17.8%)	25 (21.2%)		
Bachelor’s degree	31 (26.3%)	22 (18.6%)	22 (18.6%)	25 (21.2%)	18 (15.3%)		
Master’s degree	9 (25%)	7 (19.4%)	9 (25%)	7 (19.4%)	4 (11.1%)		
Doctorate degree	4 (40%)	2 (20%)	3 (30%)	1 (10%)	0 (0%)		
Income							
Less than $25,000	9 (18.8%)	9 (18.8%)	10 (20.8%)	12 (25%)	8 (16.7%)	22.0	0.34
$25,000–50,000	14 (15.7%)	14 (15.7%)	22 (24.7%)	23 (25.8%)	16 (18%)		
$50,000–100,000	29 (28.4%)	23 (22.5%)	14 (13.7%)	16 (15.7%)	20 (19.6%)		
$100,000–200,000	13 (18.1%)	15 (20.8%)	19 (26.4%)	10 (13.9%)	15 (20.8%)		
$200,000 or more	1 (7.7%)	5 (38.5%)	1 (7.7%)	4 (30.8%)	2 (15.4%)		
Prefer not to say	1 (8.3%)	2 (16.7%)	2 (16.7%)	4 (33.3%)	3 (25%)		

	*M*(*SD*)	*M*(*SD*)	*M*(*SD*)	*M*(*SD*)	*M*(*SD*)	*F*	*p*
	*n* = 67	*n* = 68	*n* = 68	*n* = 69	*n* = 64		
Age	32.4 (10.97)	33.9 (12.70)	36.1 (12.96)	36.5 (12.61)	39.0 (14.63)	2.60	0.038
BMI	27.3 (8.02)	27.8 (8.15)	26.0 (8.00)	27.0 (8.57)	28.3 (7.66)	0.73	0.57

*AN* Anorexia Nervosa, *AAN* Atypical Anorexia Nervosa, *MDD* Major Depressive Disorder, *BMI* Body Mass Index

### Measures/materials

#### Reported and Intended Behavior Scale (RIBS)

The RIBS [44] is an eight-item measure assessing previous experiences with others with mental illness (four items, anchored *yes, no*, and *don’t know*) and four items assessing intentions to spend time with/be close to those with mental illness in the future, rated 1 (*Disagree strongly*) through 5 (*Agree strongly*). In the current study, behavioral intention items (items 5–8; e.g., “In the future, I would be willing to live with someone with a mental health problem.”) were totaled for each participant, with higher scores indicating more favorable attitudes toward individuals with mental illness. The sample in which the measure was validated supported its test-retest reliability, consensus validity, and internal consistency [43]. In the current study, items 5–8 yielded Cronbach’s *α* = 0.84; McDonald’s ω = 0.85.

#### Community attitudes toward mental illness scale (CAMI)

The CAMI [45] is a 40-item questionnaire assessing community attitudes toward those with mental illness, rated from 1 (*Strongly disagree*) to 5 (*Strongly agree*). The CAMI has four subscales, two positively-valenced—Benevolence and Community Mental Health Ideology, and two negatively-valenced—Social Restrictiveness and Authoritarianism. An example Authoritarianism item is: *“*As soon as a person shows signs of mental disturbance, he should be hospitalized*.”* The sample in which the measure was validated supported the scale’s construct validity, internal consistency, and predictive validity. Items across all four subscales were averaged and scored such that higher scores reflected more supportive attitudes toward those with mental illness. Cronbach’s α and McDonald’s ω = 0.95 in the current sample.

#### Vignettes

Vignettes were modified from Varnado-Sullivan et al. [41] to align with our experimental conditions. In Varnado-Sullivan et al. [41], an experienced clinical psychologist wrote the vignettes based on previous stigma and mental health study vignettes and tested them on lab members and graduate students. The vignettes describe Susan, a White/Black woman with AN/AAN with “underweight”/“obesity,” respectively, or MDD (see Supplement).

#### Mental Health Stigma

The Mental Health Stigma measure was taken directly from Varnado-Sullivan et al. [41]; (Table 2) to measure participants’ perceptions of a woman, Susan, described in the vignettes. Varnado-Sullivan et al. [41] created this scale from prior mental health stigma studies. Items were measured on a 7-point Likert-type scale from 1 (*Not at all/Never)* to 7 (*Extremely/Always/Definitely*). Items stated: “I believe Susan is…” and included adjectives such as “likable” and “disciplined.” Items were averaged and all but one item were reverse scored, such that higher scores reflect greater mental health stigma, or lower scores on the positively-valenced traits. Cronbach’s α = 0.85 and McDonald’s ω = 0.83 in the current study.

**Table 2:** Means and standard deviations for mental health stigma scale without covariates

	AN white *n* = 68	AN black *n* = 68	AAN white *n* = 69	AAN black *n* = 68
1. Likable°	3.59 (0.97)	3.54 (1.11)	3.26 (1.11)	3.06 (1.09)
2. Social°	4.78 (1.42)	4.74 (1.44)	4.61 (1.46)	4.57 (1.66)
3. Interpersonally skilled°	4.34 (1.25)	4.35 (1.09)	4.34 (1.25)	3.68 (1.32)
4. Enthusiastic°	4.24 (1.38)	4.25 (1.30)	4.13 (1.38)	4.03 (1.58)
5. Driven°	2.75 (1.39)	3.24 (1.43)	2.97 (1.27)	2.71 (1.48)
6. Attractive°	3.91 (1.13)	3.99 (1.15)	4.03 (1.10)	3.84 (1.23)
7. Kind°	3.49 (1.00)	3.49 (0.91)	3.33 (0.95)	3.15 (1.15)
8. Intelligent°	3.50 (1.17)	3.65 (1.09)	3.42 (1.13)	3.15 (1.03)
9. Happy°	5.53 (1.46)	5.47 (1.37)	5.36 (1.29)	5.31 (1.48)
10. Disciplined°	2.68 (1.31)	3.29 (1.66)	3.28 (1.52)	2.99 (1.48)
11. Lazy	1.87 (0.98)	2.21 (1.30)	2.20 (1.36)	2.13 (1.34)
12. Warm°	4.12 (0.99)	4.13 (1.29)	3.88 (1.21)	3.72 (1.09)
13. Friendly°	3.91 (0.96)	3.91 (1.03)	3.62 (1.14)	3.43 (1.08)
14. Self-confident°	5.96 (1.35)	5.85 (1.50)	5.84 (1.37)	5.81 (1.37)

Items with a ° were reverse scored so that higher scores reflect greater mental health stigma (less favorable attitudes)

#### Mental health literacy

The Mental Health Literacy scale was also taken directly from Varnado-Sullivan et al. [41]; (Table 3) and rated on a 7-point Likert-type scale from 1 (*Not at all)* to 7 (*Extremely/Definitely*). Varnado-Sullivan et al. [41] modeled this scale on previous studies examining mental health literacy. It included items such as “Others will think less of her if she seeks help.” Items were averaged and some were reverse scored such that higher scores reflect greater mental health literacy. In the current study, Cronbach’s *α* and McDonald’s ω = 0.78.

**Table 3:** Means and standard deviations for mental health literacy scale without covariates

	AN white *n* = 68	AN black *n* = 68	AAN white *n* = 69	AAN black *n* = 68
1. How serious are Susan’s problems?	6.10 (0.92)	6.04 (1.06)	5.59 (1.06)	5.56 (1.44)
2. How distressing are Susan’s problems?	6.09 (0.97)	5.82 (1.21)	5.74 (1.29)	5.51 (1.53)
3. It might not be “too bad” to be like Susan? °	5.37 (1.58)	5.94 (1.49)	5.33 (1.56)	5.32 (1.78)
4. How much of a problem is Susan’s?	6.06 (0.98)	5.79 (1.22)	5.35 (1.57)	5.63 (1.66)
5. How sympathetic [are you] towards Susan?	5.62 (1.36)	5.71 (1.27)	5.75 (1.24)	5.74 (1.53)
6. Is it bad enough to seek treatment?	5.69 (1.83)	5.87 (1.83)	5.65 (1.77)	5.07 (2.24)
7. Will she get better on her own? °	5.49 (1.13)	5.32 (1.54)	4.94 (1.52)	4.85 (1.54)
8. How difficult will it be for her to get better?	5.21 (1.14)	4.96 (1.39)	4.94 (1.15)	4.76 (1.49)
9. How much worse should it get before she should seek help? °	5.94 (1.54)	5.57 (1.83)	5.70 (1.60)	5.47 (1.70)
10. Will others think less of her if she seeks help? °	6.01 (1.24)	5.53 (1.59)	5.74 (1.58)	5.72 (1.42)
11. How embarrassed should she be about seeking help? °	6.78 (0.62)	6.59 (1.05)	6.52 (1.01)	6.57 (1.03)
12. Are these normal concerns for her age? °	3.91 (2.01)	4.18 (1.60)	4.25 (2.01)	4.13 (1.94)

*Items with a* ° *were reverse scored so that h*igher scores reflect greater mental health literacy

#### Fat phobia scale short-form

This commonly-used 14-item scale [46] was selected to assess whether more weight stigma was present based on the vignettes’ weight, race, and their interaction. The Fat Phobia scale lists two pairs of adjectives and asks participants to place an “X” closest to the adjective they feel best describes their beliefs, with five response options, such as “lazy/industrious.” Items are averaged and some were reverse scored such that higher scores indicate higher weight stigma. Cronbach’s *α* = 0.84 and McDonald’s ω = 0.85 in the current study.

#### Manipulation check

After reading the vignette, participants completed three multiple choice and one open-ended question about the vignette to assess accuracy and attention in reading the vignette: “How old is Susan?”; “What race/ethnicity is Susan?”; and “What is Susan’s weight range?”. We also asked an open-ended question: “What symptoms did Susan report?”.

### Procedure

Participants were informed that they were joining a study on perceptions of people with mental health disorders. Data were collected in June 2024. The Union College Institutional Review Board determined this study to be exempt from IRB review due to being low risk as per 45 CFR 46.104(d)(3). After completing the informed consent and passing a series of captchas, participants completed demographic questions, the RIBS, and the CAMI, which were presented in a random order. Participants were then randomly assigned to one of the five vignettes, then completed a manipulation check, and the Mental Health Stigma scale, Mental Health Literacy scale, and the Fat Phobia Scale, presented in a random order. Participants were debriefed and compensated $1.80 for completing the 10-minute survey.

### Statistical analyses

Linear regression analyses were first conducted to assess whether participants’ demographic characteristics were significantly associated with the dependent variables. For significant results, MANCOVAs were run, including any significant demographic variables as covariates alongside the RIBS and CAMI; vignette weight and race were assessed as independent variables, and the mental health stigma assessment and mental health literacy scale were assessed as dependent variables, with ANCOVA used to assess the Fat Phobia Scale as a dependent variable.

Because previous research has demonstrated significant correlations between BMI and internalized weight bias (e.g., r = 0.32, p < 0.001) [47], between BMI and externalized weight bias [48, 49]; and between the Fat Phobia scale and measures of internalized weight bias [50, 51], we collected height and weight to calculate participants’ BMI, to control for variance in the dependent variables that was attributable to participants’ BMI.

Analyses were conducted for all participants and for participants who correctly answered the weight and race multiple choice manipulation check questions.

## Results

### Manipulation check

Overall, participants were attentive to the details of the vignettes, with 75.5% correctly identifying the vignette’s age, and 83.6% in the experimental conditions correctly identifying both her weight and race. The pattern of results did not differ across analyses for participants who answered all manipulation check items correctly and those who missed one or more of those questions. Consequently, we reported results from all participants in experimental conditions.

### Mental health stigma

No demographic variables significantly predicted mental health stigma, so none were included as covariates. A 2 (vignette weight) x 2 (vignette race) MANCOVA was conducted with the 14 mental health stigma items as dependent variables. The CAMI scale was statistically significant, *F* (14, 254) = 3.38, *p* < 0.001, *η*^2^ = 0.16, as was vignette weight, *F* (14, 254) = 1.81, *p* = 0.037, *η*^2^ = 0.091. Contrary to expectations, RIBS, *F* (14, 254) = 0.87, *p* = 0.60, *η*^2^ = 0.046, vignette race, *F* (14, 254) = 0.49, *p* = 0.94, *η*^2^ = 0.026, and Vignette Weight x Race interaction, *F* (14, 254) = 1.02, *p* = 0.44, *η*^2^ = 0.053, were not statistically significant.

Examining Mental Health Stigma items individually, after controlling for covariates, vignette weight significantly predicted Item 1 “Likable,” *F*(1, 267) = 9.67, *p* = 0.004, *η*^2^ = 0.031, Item 3 “Interpersonally Skilled”,* F*(1, 267) = 10.28, *p* = 0.009, *η*^2^ = 0.025, and Item 8 “Intelligent,” *F*(1, 267) = 4.21, *p* =. 041, *η*^2^ = 0.016, with higher-weight women perceived as more likable, less interpersonally skilled, and more intelligent. Vignette race did not significantly predict any items, but the Race x Weight interaction significantly predicted Item 5 “Driven,”* F*(1, 267) = 5.42, *p* = 0.021, *η*^2^ = 0.020 and Item 10 “Disciplined,”* F*(1, 267) = 6.81, *p* = 0.010, *η*^2^ = 0.025, with the Black woman with AAN and White woman with AN rated as most driven and disciplined, and the Black woman with AN rated as least driven and disciplined, with no differences in discipline between the Black woman with AN and White woman with AAN. Means and standard deviations for the mental health stigma items without covariates are presented in Table 2.

### Mental Health literacy

Participants’ gender and race/ethnicity significantly predicted the mental health literacy scale, so they were included as covariates, alongside the RIBS and CAMI. A 2 (vignette weight) x 2 (vignette race) MANCOVA was conducted with the 12 Mental Health Literacy scale items as dependent variables. The CAMI, *F* (12, 254) = 7.75, *p* < 0.001, *η*^2^ = 0.27, was significantly associated with Mental Health Literacy scores, as was vignette weight, as hypothesized, *F* (12, 254) = 2.65, *p* = 0.002, *η*^2^ = 0.011. Analyses did not reveal significant results for the RIBS, *F* (12, 254) = 0.46, *p* =.94, *η*^2^ =.021, vignette race, *F* (12, 254) = 1.31, *p* = 0.21, *η*^2^ = 0.058, or for Vignette Weight x Race interaction, *F* (12, 254) = 0.99, *p* = 0.46, *η*^2^ = 0.045. Means and standard deviations for the Mental Health Literacy scale without inclusion of covariates can be seen in Table 3.

Examining Mental Health Literacy items individually, after controlling for covariates, vignette weight significantly predicted Item 1, “How serious are Susan’s problems?,” *F*(1, 265) = 18.89, *p* < 0.001, *η*^2^ = 0.067; Item 2, “How distressing are Susan’s problems?”, *F*(1, 265) = 7.76, *p* = 0.006, *η*^2^ = 0.028; Item 3 “It might not be ‘too bad’ to be like Susan”,* F*(1, 265) = 4.53, *p* = 0.034, *η*^2^ = 0.017, Item 4, “How much of a problem is Susan’s?,” *F*(1, 265) = 10.64, *p* = 0.001, *η*^2^ = 0.039; Item 6, “Is it bad enough to seek treatment?”, *F*(1, 265) = 3.93, *p* = 0.049, *η*^2^ = 0.15; and Item 7 “Will she get better on her own?,” *F*(1, 265) =10.24, *p* = 0.002, *η*^2^ = 0.037. Across all six of these items, participants reported that Susan had a more significant problem when she was described “underweight” than when she was described as having “obesity.” Vignette Race and Weight x Race interactions were not significant for any individual Mental Health Literacy items.

### Weight stigma

No demographic variables were significantly associated with Fat Phobia Scale scores, so only RIBS and CAMI were included as covariates. A 2 (vignette weight) x 2 (vignette race) ANCOVA was conducted with the Fat Phobia scale as the dependent variable. As hypothesized, analyses revealed a significant Vignette Weight x Race interaction, *F* = 6.63, *p* = 0.014, *η*^2^ = 0.023, as well as the CAMI, *F* = 6.91, *p* = 0.009, *η*^2^ = 0.026. In contrast, we did not find significant effects of vignette weight, *F* = 0.48, *p* = 0.49, *η*^2^ = 0.002, vignette race *F* = 0.15, *p* = 0.70, *η*^2^ = 0.001, or RIBS, *F* = 0.27, *p* = 0.61, *η*^2^ = 0.001, on Fat Phobia Scale scores. Weight stigma was higher for the Black low-weight woman and the White high-weight woman. Means and standard deviations for the Fat Phobia scale can be seen in Table 4.

**Table 4:** Means and standard deviations for the fat phobia scale

	AN	AAN
Black	2.86 (0.06)	2.75 (0.06)
White	2.71 (0.06)	2.83 (0.06)

Several items were reverse scored prior to averaging such that higher scores indicate more weight stigma

## Discussion

To our knowledge, previous research has not examined the influence of both weight and race and their interaction on mental health literacy, mental health stigma, and weight stigma. We hypothesized that participants would view the vignette case, Susan, as less in need of treatment, showing more mental health and weight stigma when she was described as having “obesity” compared to “underweight” [19]. Additionally, we hypothesized that greater mental health stigma and weight stigma would be present for the Black women in both the AN and AAN conditions than the White women in both conditions [32, 42, 43], but that the Black woman described as being in the “obese” weight category would experience the highest stigma of the four conditions. Results partially supported our hypotheses.

Aligned with our hypotheses, based on prior research that varied vignettes’ weight [15, 18, 19], we found significant main effects of vignette weight on perceptions of the Susan’s personality traits (Mental Health Stigma items and Fat Phobia Scale) and on perceptions of how serious Susan’s problems were (Mental Health Literacy items). Mental health stigma occurred, however, in an unexpected pattern. Higher weight women were perceived as more likeable and intelligent, but less interpersonally skilled, in contrast to expectations. The significant main effects of weight on participants’ mental health literacy scores were as expected: they rated Susan’s problem as more serious, distressing, as more of a problem, as being bad enough to need treatment, and as less likely to get better on her own when she was described as “underweight” compared to when she was described as being in the “obese” weight range. Thus, this finding contributes to a broader literature suggesting that larger-bodied individuals with eating disorders are less likely to be diagnosed by healthcare providers [8, 17], mental health care providers [18], and are also less likely to be identified by the public as needing ED treatment [17]. Consequently, those in larger bodies may be less likely to perceive a need for treatment themselves, may not be pushed by loved ones to seek treatment, and may receive less support if they do seek treatment.

There were no significant main effects of race on participants’ mental health stigma, weight stigma, or perceptions of the severity of Susan’s problem, contrary to our expectations. This is, to our knowledge, the first study that manipulated a vignette’s race to determine whether it affected mental health stigma, weight stigma, or people’s recognition of the severity of an ED. Additionally, in contrast to hypotheses, there was no intersection of weight and race in mental health stigma or literacy scales overall, with only two mental health stigma items significantly impacted. The Black woman with AAN and White woman with AN were perceived as most driven and disciplined, and the Black woman with AN was rated as the least driven and disciplined.

In line with our expectations, there was a significant interaction between the vignette’s weight and race on weight stigma. However, in contrast to our expectations that negative perceptions would be greatest for a Black, larger-bodied woman, our findings suggest that the most negative perceptions occurred when the vignette described either a larger-bodied White woman or an “underweight” Black woman. Thus, it largely appears that people’s intersectional stigma is greatest when individuals do not fit with societal stereotypes about who can or should have what type of body. This may be explained by different body ideals for Black women, and restrictive eating presenting differently in Black women than in White women, which many ED screens fail to capture [52]. Black women may also pursue the thin ideal for different reasons than White women [52].

Additionally, participants’ intentions to interact with those with mental illness did not impact their perceptions of the vignette across any of our outcome variables, but their pre-existing positive attitudes towards those with mental illness were significant predictors of more favorable views towards the vignettes overall, lower weight stigma, and higher mental health literacy.

Compared to prior research [31, 41], the current study aimed to recruit a more diverse sample in terms of age, education, and geographic location within the U.S. by using online recruitment, adding to the generalizability of our results. We also controlled for demographic factors in our analyses. Mental health literacy scores significantly varied by demographic factors, namely race (Supplementary Material 2). While our results suggest evidence of weight discrimination, with some evidence suggesting more negative attitudes in particular towards White larger-bodied women with AAN and Black women with AN, we hesitate to make conclusions regarding a lack of racial bias in those with EDs in larger bodies, based on the preponderance of literature suggesting racial- and weight-based discrimination [53] remain prevalent within US society, typically with more negative attitudes towards REM individuals across a range of settings (e.g.,[54–56]). Future research should examine weight and race stigma and their intersection in diverse participants, and in other countries and regions with different racial stereotypes and tensions, to better understand the intersections between weight- and race-based stigma. Additionally, it would be helpful to assess participants’ political and social ideology, to determine whether these factors play a role in weight stigma, mental health stigma, and mental health literacy. There may also be notable cultural and geographic differences in how individuals express weight, race, and mental health stigma; for example, individuals from Western countries have been found to exhibit more mental health stigma toward minoritized groups that have historically and presently experienced systemic disadvantages, discrimination and prejudice, marginalization, and reduced access to power and resources compared to the dominant group [57, 58]. Thus, future research in this field may also benefit from implicit assessments of stigma.

### Limitations

The current study is not without limitations. Participants read a vignette about a person with mental health concerns (e.g., AN/AAN, MDD) in an online platform, but may not have been able to personalize these vignettes. Future research may use video vignettes to confirm our results. Despite our limitations, this study also had notable strengths.

### Implications and conclusions

The current study examined how weight and race interact to influence mental health stigma and mental health literacy pertaining to AN and AAN in Black and White women. It was hypothesized that participants would view the woman in the vignettes as having a less serious problem, being less in need of treatment, and having less favorable attitudes toward her when she was described to be “obese” compared to “underweight,” and when we she was described as being Black compared to the White women, with the Black “obese” woman with AAN stigmatized to the greatest extent. The data somewhat supported these hypotheses, largely for the weight stigma outcome, but overall, White larger-bodied women and low-weight Black women were stigmatized the most. Future research should extend literature on mental health stigma and mental health literacy among different eating and mental health disorders by examining how factors such as race, sexual orientation, and gender identity of the vignette influence these perceptions across diagnoses. Research suggests that intersections of marginalized identities often increase stigma [59]), although this was not uniformly the case in the current study. Future research should also aim to understand stigma origins to help reduce and prevent bias, especially in higher-weight persons and individuals with intersecting marginalized identities.

## Supplementary Information

Supplementary file 1

## Data Availability

Data that support the findings of this study are available from the corresponding author upon request and will be made publicly available from https://osf.io/mv6bj/?view_only = deb7f929 d2e2404589cfb7 d5ea3e4514 upon publication of this manuscript.

## Footnotes

1 The term “obese” was used in the vignettes because it is still commonly used and would be best understood by participants. In the manuscript, the term obese/obesity is used only when referring to language used in other studies or the vignette description, due to its association with stigma for those in larger bodies.

2 “Racial and ethnic minority” is used here as a term not referring to the number of people in a population, but their social positioning within our society, specifically to describe groups that have historically and presently experienced systemic disadvantages, discrimination and prejudice, marginalization, and reduced access to power and resources compared to the dominant group. We use both race (socially constructed category) and ethnicity (cultural heritage and identity) because both social positioning (race) and identity (ethnicity) intertwine and influence someone’s lived experiences (perception of self and of the self, relative to society). As it relates to the current study, body image and eating disorder research has notoriously omitted racial and ethnic minority individuals, such that we have a less nuanced and less complete understanding of body image and eating among racial and ethnic minority individuals [47]. However, it is important to recognize that these individuals are not monolithic and that their body image and eating experiences are varied and heterogeneous.
