# Supplementary material for: The impact of weight and race on perceptions of anorexia nervosa: a replication and extension of Varnado-Sullivan et al. (2020)
Source: Eat Weight Disord. 2025 Apr 30;30(1):39. doi: 10.1007/s40519-025-01748-x (PMC12043770; doi:10.1007/s40519-025-01748-x)
Supplement: Supplementary file 1 [file 40519_2025_1748_MOESM1_ESM.docx]

**Supplementary Files**

**Eating Disorder Vignettes**

Susan, an **18 year old White/Black female**, has just started her first year of college. She is 5’8” tall and weighs 110 lbs./200 lbs **[16.7 BMI/30.4 BMI]**, which places her in the **underweight/obese range** for her age. However, she is extremely afraid of gaining weight because she really does not want to “be fat.” She knows that to be happy, she just needs to lose a few more pounds. She began dieting several years ago, initially by avoiding anything “fatty” or junk food. Susan was able to lose quite a bit of weight over time. Now, she will only eat very small portions of “healthy” foods. Susan exercises every day for several hours by running or doing aerobics. Whenever Susan feels like she has eaten too much, she will exercise extra to help burn the calories. She feels cold a lot of the time and often has stomach trouble. She has not had her menstrual cycle for a long time. Even though others tell her they are worried about her, she thinks she is just being healthy. Susan does not go out with her friends anymore, because she does not want to eat in front of them. She spends a lot of time on the internet, looking at websites that have information about dieting, support and tips from others who want to lose weight, and images she can use for “thinspiration.”

Control:

Susan is an **18 year-old** woman who has just started her first year of college. She is 5’8” tall and weighs 135 lbs., which places her in the **normal weight range** for her age. For the past few weeks, she has been feeling unusually sad and does not enjoy doing anything. She feels like this all of the time, pretty much the whole day, every day. She has not had much of an appetite and has not been eating well. Susan has felt this way before, and it lasted for a while. Now, she is worried that this will not go away again. Susan does not like herself much, and always feels guilty about something. Whenever Susan tries to read or watch TV, she cannot concentrate and cannot explain anything about the plot. She feels “blah” a lot of the time and often feels very achy. She is always very tired, but cannot fall asleep at night. Other people have noticed how much she has changed, and her friends and family are very worried. Susan does not go out with her friends much anymore, because she usually does not feel like being around them.

**Table S1 A**

*Linear Regression Analysis for Demographic Factors and Mental Health Stigma*

| Effect | Unstandardized  Estimate | *SE* | *ß* | 95% CI | | *p* |
| --- | --- | --- | --- | --- | --- | --- |
|  |  |  |  | L.L. | U.L. |  |
| Intercept | 3.55 | 0.22 |  | 3.13 | 3.98 | <.001 |
| Age | -0.001 | 0.004 | -0.13 | -0.01 | 0.006 | .83 |
| Gender | -0.03 | 0.05 | -0.05 | -0.12 | 0.06 | .45 |
| Race/Ethnicity | 0.03 | 0.03 | 0.07 | -0.02 | 0.08 | .24 |
| BMI | 0.01 | 0.01 | 0.13 | 0.000 | 0.02 | 0.04 |

*Note.* The linear regression for the full model for mental health stigma was not statistically significant, *F* (4, 268) = 1.46, *p* = .21.

**Table S1 B**

*Linear Regression Analysis for Demographic Factors and Mental Health Literacy*

| Effect | Unstandardized  Estimate | *SE* | *ß* | 95% CI | | *p* |
| --- | --- | --- | --- | --- | --- | --- |
|  |  |  |  | L.L. | U.L. |  |
| Intercept | 5.76 | 0.23 |  | 5.31 | 6.21 | <.001 |
| Age | -0.003 | 0.004 | -0.04 | -0.01 | 0.005 | .51 |
| Gender | -0.07 | 0.05 | -0.09 | -0.16 | 0.02 | .14 |
| Race/Ethnicity | -0.09 | 0.03 | -0.20 | -0.14 | -0.04 | <.001 |
| BMI | 0.006 | 0.006 | 0.06 | -0.007 | 0.02 | .36 |

*Note.* The linear regression for the full model for mental health literacy was statistically significant, *F*(4, 268) = 3.60, *p* = .007.

**Table S1 C**

*Linear Regression Analysis for Demographic Factors and the Fat Phobia Scale*

| Effect | Unstandardized  Estimate | *SE* | *ß* | 95% CI | | *p* |
| --- | --- | --- | --- | --- | --- | --- |
|  |  |  |  | L.L. | U.L. |  |
| Intercept | 2.65 | 0.12 |  | 2.41 | 2.89 | <.001 |
| Age | -0.002 | 0.002 | -0.07 | -0.01 | 0.002 | .24 |
| Gender | 0.01 | 0.03 | 0.02 | -0.04 | 0.06 | .74 |
| Race/Ethnicity | 0.02 | 0.01 | 0.07 | -0.01 | 0.04 | .27 |
| BMI | 0.003 | 0.003 | 0.06 | -0.003 | 0.01 | .30 |

*Note.* The linear regression for fatphobia was not statistically significant, *F*(4, 268) = 1.01, *p* = 0.36
